# Factors affecting adoption of self-service E-ticketing technology: A study on heritage sites in Bangladesh

**DOI:** 10.1016/j.heliyon.2023.e14691

**Published:** 2023-03-20

**Authors:** Md Nurnobi Islam

**Affiliations:** Department of Marketing, Begum Rokeya University, Rangpur, 5404, Bangladesh

**Keywords:** E-ticketing, Heritage visitors, Technology acceptance model, Smart-PLS, Privacy concern, Ease of use, Perceived usefulness, Attitude, Intention

## Abstract

E-ticketing as a self-service technology has become very popular in tourism, especially in the airline industry. But, the e-ticketing adoption behavior of heritage visitors received very little attention from tourism researchers, especially in developing countries. This research applied the technology acceptance model and theory of planned behavior to investigate heritage visitors' self-service e-ticketing adoption behavior. This research used primary data collected from visitors at five heritage sites in Bangladesh. The PLS-based structural equation modeling technique was applied to test the structural model. This research has found that ease of use and subjective norm have significant positive associations with attitude and intention to use e-ticketing. But, privacy concern has a significant negative association with attitude and e-ticketing intention. Perceived usefulness has a significant positive association with attitude but not e-ticketing intention. Attitude plays an important role in e-ticketing adoption behavior by moderating the relationships between e-ticketing intention and other constructs (ease of use, subjective norm, and privacy concern). This research provides valuable insights into visitors' technology adoption behavior for the academics and authorities of heritage sites in developing countries. This paper has discussed the theoretical and practical implications.

## 1 Introduction

Modern information and communication technologies play significant roles in our daily life. Technology has altered how we live, work, play, and travel [1]. With its widespread use in every sector, technology is becoming a necessity rather than a complementary tool [2]. Tourism destination marketers are no exception in adopting advanced technologies [3]. Technological advancement has changed how marketers design their self-services and how customers use them [4]. Technologies are now used in customer service, online transactions, and customer self-service [5,6]. The advent of the internet and e-commerce technologies has contributed to this paradigm shift in sales and marketing in the tourism sector.

Technology offers advantages to tourism businesses and their customers. Technology enhances organizational competitiveness by bringing efficiency to business operations [7]. Travel businesses can bypass the intermediaries to directly interact with their customers using e-commerce technologies and create extra revenues [8]. Information technology brings marketing efficiency by reducing the distribution costs of business [9]. Self-service technologies enhance customer involvement in service delivery [6] and improve customer satisfaction by saving time in booking and check-in procedures [10]. However, not all tourists are innovative in adopting new technologies because their need for distinctiveness and assimilation varies [11]. And not all travel businesses can leverage the technologies equally to create value for their technology-savvy customers [12].

Addressing cognitive, emotional, and contextual issues is necessary for successfully assisting technology adoption [13]. According to Rogers and Murcott [14], the characteristics of users, the attributes of innovations, the type of innovation decision, channels of communications, the nature of the social system, and promotional efforts are associated with the speed with which society accepts an innovation. Ease of use and perceived usefulness are essential for adopting new technology [15]. Complex and less helpful technology takes longer to get accepted by most members of society. Organizational reputation, which reduces perceived risk and instills trust in customers, is also conducive to adopting e-services [16]. In the post-pandemic period, the demand for augmented reality, artificial intelligence, robotics, and other contactless technologies that provide safety and a memorable experience may rise [17,18]. Adopting innovative strategies, including smart technologies, will help faster recovery and adapt to post-pandemic challenges.

E-ticketing as a self-service technology has become very popular in tourism, especially in the airline industry. Convenience and ease of use are the reasons customers choose e-ticketing [19]. E-ticket increases convenience as travelers do not need to carry paper tickets; they can buy them online anytime. Moreover, e-ticket minimizes the risk of mishandling tickets, such as destroying or losing them. But, as the customers share their personal information in the e-ticketing system, e-ticketing involves privacy risks. Data privacy has become a key concern for government, industries, tourists, and tourism service providers, given the use of personal data for commercial purposes and political control [20]. There is concern that new technologies undermine privacy rights by facilitating the collection, storage, processing, and combination of personal data that government agencies and businesses use without customers' consent [21]. Lowering the perceived risk is important to promote trust, perceived service quality, and intention to use e-services [16].

Existing research on e-ticketing adoption behavior has widely focused on the airline industry [1,8,9,19,22]. Research on self-service technology adoption behavior, specifically the e-ticketing adoption behavior of heritage visitors, is limited. As a result, we don’t know how heritage visitors in a developing country like Bangladesh respond to self-service technology like an e-ticketing system. Most of the heritage destinations in Bangladesh have not yet adopted an e-ticketing system. These destinations sell paper tickets. These traditional practices increase staffing, paper, and printing costs but sometimes increase visitor discomfort due to lining up to buy a ticket at the booth. Transforming this ticketing practice will save operating costs of the heritage sites and improve visitors' convenience. However, how the visitors, mostly domestic visitors, will respond if the heritage tourism destinations switch from these traditional practices to a self-service e-ticketing system need to be known. Research is, therefore, required to investigate heritage visitors' behavioral intention to use the e-ticketing system. Understanding the e-ticketing acceptance behavior of heritage visitors is essential to digitally transform the ticketing system, improve the quality of visitor experience and ensure faster acceptance of the self-service technology in heritage sites in developing countries.

Drawing on the technology acceptance model [15,23,24] and the theory of planned behavior [25,26], this study has hypothesized and tested an integrated model to explain various factors associated with heritage visitors' intention to use the e-ticketing system in Bangladesh. The role of privacy concerns has also been tested. The findings will help the heritage authorities in developing countries implement strategies to increase visitors' adoption of e-ticketing and other self-service technologies.

## 2 Literature review

### 2.1 Theoretical background

The technology acceptance model (TAM) is one of the most influential models used to explain users' information systems or technology acceptance behavior. Fred Davis [27] first introduced TAM in his doctoral thesis. This original model has four key components: perceived usefulness, perceived ease of use, attitude, and actual use. This model proposed that perceived usefulness and ease of use are two critical determinants of attitude predicting the actual use of an information system. The original TAM was prepared based on a theory explaining consumer behavior, known as the theory of reasoned action [28]. The theory of reasoned action (TRA) explains that a person’s actual behavior is determined by his behavioral intention, which is influenced by his attitude towards the behavior and subjective norm. Davis et al. [23] adapted the original TAM by including intention as a determinant of the actual usage of computer systems. The final version of TAM eliminated the attitude construct based on the result that perceived usefulness and ease of use directly influence the behavior intention [24]. Since then, much research has modified, extended, applied, and criticized the model [23,[29], [30], [31], [32]]. Venkatesh and Davis [30] extended the original model and proposed TAM2, which incorporated the subjective norm as an additional determinant of usage intention. TAM2 theorized that subjective norm, image, job relevance, and result demonstrability are determinants of perceived usefulness.

After a few years, Venkatesh et al. [31] proposed a unified theory of acceptance and use of technology (UTAUT) after analyzing eight popular theories of technology acceptance and consumer behavior. UTAUT consists of four determinants of intention and usage: performance expectancy, effort expectancy, social influence, and facilitating conditions such as organizational readiness, infrastructure adequacy, etc. These determinants are somewhat similar to TAM2 [30]. For instance, performance expectancy is similar to perceived usefulness, effort expectancy is similar to the ease of use, and social influence is somewhat similar to the subjective norm [31,33]. Later on, Venkatesh et al. [32] extended the UTAUT by adding three more constructs, i.e., hedonic motivation, price value, and habit, as predictors of behavioral intention. The authors of the TAM or extended TAM based their studies on the employees of organizations [27,30,31], MBA students [23,24], and potential customers [32] to investigate the adoption behavior of information systems or computer systems. But, other researchers used TAM [23] to investigate the acceptance or rejection of self-service technologies or e-ticketing in the tourism sector [34,35]. Calantone et al. [29] criticized that the western model predicting technology acceptance behavior did not fit in the context of a developing country like China.

The theory of planned behavior (TPB) is another prominent model of consumer behavior [25,26]. It was developed by extending the TRA, which did not include perceived behavioral control as a predictor of behavioral intentions [28]. But, TPB proposed perceived behavioral control, in addition to attitude and subjective norm, as an important predictor of behavioral intentions. Both TRA and TPB assume that intention is the immediate determinant of actual behavior. Taylor and Todd [36] extended the TPB by decomposing its constructs into belief-based indirect measures. The authors hypothesized that relative advantage, compatibility, and complexity are related to attitude, normative influences are related to the subjective norm, and efficacy and facilitating conditions are related to perceived behavioral control. Decomposing belief structures into multi-dimensional constructs provides a comprehensive understanding of the relationships among constructs [36]. TPB [26] and decomposed theory of planned behavior [36] were used to predict consumer acceptance of self-service technologies and e-ticketing in the tourism sector [1,22,37]. Other researchers integrated constructs of TPB and TAM to predict customers' technology acceptance behavior [5,38].

This research has integrated the final version of TAM [24] and the TPB [26] to investigate the self-service e-ticketing acceptance behavior. TAM [24] is easy to understand compared to the subsequently modified versions [30,31] of this model and yet has shown high predictive capacity in many contexts. Although visitors usually visit the sites as groups, the final modified version of TAM [24] did not include subjective norms and attitudes. Considering other group members' influence on their decisions, this research has adapted subjective norms and attitudes from TPB [26].

### 2.2 Behavioral intention

Warshaw and Davis [39] defined intention as the extent to which an individual has prearranged plans to engage or not in a specified future behavior. Intentions indicate how hard individuals are willing to try and how much effort they plan to exert to perform a given behavior [40]. It is often confused with but different from motives. The intention is what a person aims at or chooses; the motive determines the aim or choice. Intention can be a cause or reason, depending on the circumstances [41]. It is closely related to mental states, especially beliefs about the future and the agent’s abilities [42]. An individual's intention is the immediate determinant of an action [25,28]. First, a person forms an intention to perform a particular behavior. Then, intention causes the person to engage in the behavior. Normative beliefs or cognitive factors, such as attitude, perceived social influence, and perceived behavioral control, have influences on intention [25,26,28,43]. However, when individuals lack control over their behavior, when there is a chance for social reaction, and when the environment is favorable to habit formation, intentions have less of an impact on actual behavior [44]. The intention to use technology is a function of perceived ease of use and usefulness [24,30]. In this research, intention refers to the heritage visitors' intention to use an e-ticketing system for their heritage tours.

### 2.3 Attitude

There are two popular views on attitude-functional and constructive views. The functional theory views attitude as memory-based, suggesting that consumers develop their attitudes following their initial exposure to stimuli, which they then store in memory [45]. Consumers can recall their attitudinal reactions from memory when prompted by marketers, advertisers, or researchers. According to functional theory, attitude is a pre-disposed tendency to respond to an object favorably [46]. Attitude serves four different functions: knowledge, value-expressive, social-adjustive, and utilitarian functions [[47], [48], [49]]. Individuals form attitudes to organize, structure, and summarize large amounts of information about an object and thus serve the knowledge function [48]; attitudes express values, preferences, and perceptions of people to others and thus serve a value-expressive function [50]; attitudes enable people to live up to others' expectations, facilitating effective and seamless social engagement and thus serve a social-adjustive function [51]; attitudes summarize rewards and punishments obtained from an object and thus serve a utilitarian function [52]. Contrarily, the constructivist considers attitude evaluative judgments [45,53]. According to Ajzen [25], attitude is an individual's positive or negative evaluation of performing a given behavior. The constructive theory assumes that consumers do not recall their attitudes from memory but instead calculate immediately in accordance with their contextual goals [54].

According to TPB, attitude determines intention [25,26]. The TAM also hypothesized attitude as a function of both perceived ease of use and perceived usefulness of a system and as a significant determinant of actual system use [27]. A positive attitude toward technology is important to accept technology [55]. Park [56] investigated the role of attitude in explaining behavioral intention from cross-cultural perspectives and found that attitude is a significant predictor of behavioral intention both in collectivist and individualistic cultures. This research aims to measure the extent to which attitude toward technology is associated with behavioral intention to use a self-service e-ticketing system. Therefore, H1 is proposed:

H1 Attitude has a significant positive association with e-ticketing intention.

### 2.4 Ease of use

Perceive *ease of use* refers to the extent to which a person believes that using a particular system would be free of effort [39]. Perceived ease of use is similar to Bandura's self-efficacy theory [57]. However, Venkatesh and Davis [24] suggested that computer self-efficacy enhances the perceived ease of use of a system before and after the use of a system. The authors suggested that training interventions to increase user efficacy is more effective than interface design in enhancing user adoption of technology. Self-efficacy helps mitigate the anxiety associated with technology use and enhances the perceived ease of use and usefulness, both of which are associated with the usage or acceptance of technology [24,39,58]. TAM proposed perceived ease of use as a determinant of attitude toward an information system. Ease of use is also a determinant of intention to use technology [15,24]. The research found that perceived ease of use is related to heritage visitors' intention to use new technology for their heritage tours [59]. Therefore, H2 and H3 are proposed:

H2 Perceived ease of use has a significant positive association with attitude toward e-ticketing.

H3 Perceived ease of use has a significant positive association with e-ticketing intention.

### 2.5 Perceived usefulness

Perceived usefulness refers to the extent to which a particular technology enhances users' performance [39]. Perceived usefulness is essential for users to adopt new technology. Perceived ease of use of an information system or new technology is associated with perceived usefulness. Perceived usefulness is a determinant of attitude [15,39] and intention to adopt an information system or new technologies [15,24,30,39]. Several factors, including subjective norm, image, job relevance, output quality, and result demonstrability, are also associated with the perceived usefulness of technologies [30]. Chung et al. [59] found that perceived usefulness is associated with heritage visitors' intention to use new technology, such as augmented reality, at heritage sites. Therefore, H4,H5, and H6 are proposed:

H4 Perceived usefulness has a significant positive association with attitude towards e-ticketing.

H5 Perceived usefulness has a significant positive association with e-ticketing intention.

H6 Ease of use has a significant positive association with the perceived usefulness of e-ticketing.

### 2.6 Subjective norm

Social pressure and the influence of others' presence have been linked to more prosocial decisions [60]. Allport [61] demonstrated that a subtle cue, such as the perceived presence of others, might have a similar impact even though it was unrelated to actual observation and potential future gain. Subjective norm is a person's beliefs that individuals or groups who are important to him might think he should or should not perform the behavior [25,26]. According to Kelman [62], individuals adjust their attitudes and behavior in response to their perception of what others might say or do. People's perception of social pressure varies across cultures. Research shows that members of a collectivist society score higher on subjective norms than those of an individualistic society [56]. Trafimow and Fishbein [63] suggested that people consider subjective norms more critical when the behavior they intend to perform involves groups than the individual alone. Subjective norms have become more influential due to technological development such as the internet [1]. People are now more influenced by the comments of others in internet-based groups or social media groups. Subjective norm is an important determinant of behavioral intention [25,26]. Individuals take subjective norms into account while changing their feelings and behaviors after engaging with people viewed as comparable, desirable, and knowledgeable [64]. Kim et al. [65] found that subjective norm does not affect behavioral intention directly. Rather, attitude mediates the relationship between subjective norms and behavioral intention. Venkatesh and Davis [30] proposed subjective norm as a determinant of intention to use technology. Therefore, H7 and H8 are proposed:

H7 Subjective norm has a significant positive association with attitude towards e-ticketing.

H8 Subjective norm has a significant positive association with e-ticketing intention.

### 2.7 Privacy concerns

The diffusion of technologies often exacerbates users' privacy concerns [21]. Consumers of the online marketplace are aware of their privacy rights. They want to control what personal information is disclosed about them, to whom, and how it will be used [66]. Privacy is an area of crucial concern in tourism because tourists' mobility across multiple jurisdictions has different privacy laws, different provisions for customer services, data storage, and other issues [20]. Privacy concerns are the most significant barrier to popularizing e-ticketing services in developing countries [19]. Privacy and technology are interrelated in that technology affects an individual's understanding of privacy, and people's knowledge and experience of privacy are necessary for wider acceptance of technology [21]. Consumers' privacy concerns are negatively associated with purchasing tickets from sources such as vendors [1]. Privacy concerns also moderate the negative relationship between attitudes toward technology and the intention to use the technology [67]. The privacy concerns of technology users need to be mitigated to increase the acceptance of technology [68]. But, the privacy concerns of heritage visitors were often neglected in heritage tourism research, especially in the context of developing countries. As a result, the degree of privacy concerns of heritage visitors and the influence of privacy concerns on their attitude and behavioral intention to accept new technologies remain unknown. Therefore, H9 and H10 are proposed:

H9 Privacy concern has a significant negative association with the attitude toward e-ticketing.

H10 Privacy concern has a significant negative association with e-ticketing intention.

**Fig. 1:**
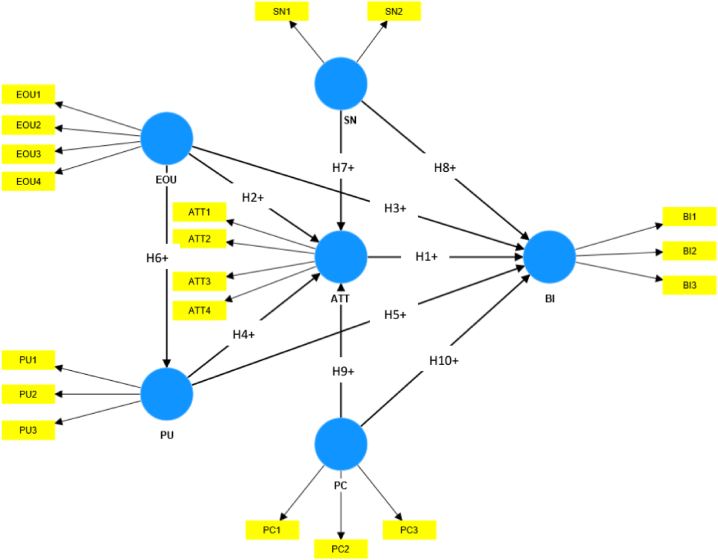
The conceptual framework.

Fig. 1 shows the conceptual framework of this research.

## 3 Research method

### 3.1 Measures

The constructs and associated indicators of the research model were adapted from previous literature. The constructs are e-ticketing behavioral intention, attitude, ease of use, perceived usefulness, subjective norms, and privacy concerns. All of them are reflective constructs. 5 points Likert scale was used to measure the responses to these indicators, with 1 being strongly disagreed and 5 strongly agreed. Both English and Bengali languages were used in the questionnaire. Each statement in the questionnaire started in English, and its Bengali translation (inside bracket) was used for respondents to understand the questionnaire clearly. The researcher took help from an academic, having expert knowledge in both languages, for the translation services. Table 1, presents the constructs and the indicators used to measure them.

**Table 1:** Indicators used to measure the constructs.

Construct	Indicators	Contents	Source
**Behavioral Intention (BI)**	BI1	I intend to use the E-ticketing system for my visit to heritage sites in the future	[35,40]
	BI2	I plan to purchase an online ticket for my visit to heritage sites in the next 6 months	
	BI3	I will use E-ticketing for my visit to heritage sites in the next 6 months	
**Attitude (ATT)**	ATT1	The E-ticketing system is enjoyable	[35]
	ATT2	The E-ticketing system is pleasant	
	ATT3	The E-ticketing system is good	
	ATT4	The E-ticketing system is useful	
**Ease of use (EOU)**	EOU1	It would be easy to learn the E-ticketing process	[15]
	EOU2	It would be easy to buy an E-ticket for my visits to heritage sites	
	EOU3	An E-ticketing system would be understandable	
	EOU4	An E-ticketing system would be flexible for my heritage tour	
**Perceived usefulness (PU)**	PU1	An E-ticketing system in heritage sites would help buy tickets more quickly	[15]
	PU2	E-ticketing in heritage sites would make it easier to buy the tickets	
	PU3	E-ticketing would be useful for me in my future heritage sites	
**Subjective norms (SN)**	SN1	Most people (who are important to me) will approve if I use an e-ticketing system for my visit to heritage sites	[35]
	SN2	Most people (who are important to me) will think that I should use an e-ticketing system for my visits to heritage sites	
**Privacy concerns (PC)**	PC1	I am anxious about the consequences of sharing my information with an E-ticketing system	[1]
	PC2	It is important to me that the heritage authority protect my identity in the E-ticketing system	
	PC3	I believe my personal information would be well-protected in the E-ticketing system of the heritage sites	

### 3.2 Data collection and analysis

Data were collected between May 2022 and June 2022. Structured questionnaires (print) were used to collect data from visitors through in-person surveys at 5 popular heritage sites in Bangladesh. The sites' visitors are considered to be the representative groups of the heritage visitors in Bangladesh. The selected heritage sites: Tajhat Jamidar Bari, Paharpur Buddhist Vihara, Mohasthan Garh, Ramsagor Dighi, and Kantojir Temple are the top tourist destinations in the northern part of Bangladesh. Data were collected from visitors inside the sites. 10 graduate-level students were trained to collect the data. Before collecting data, the researcher tested the questionnaire on 10 visitors at Tajhat Jamidar Bari. As no issue regarding the questionnaire was found, the researcher used the questionnaire for final data collection. As the sampling frame was unavailable, the random sampling technique could not be used. Respondents were selected based on the convenience sampling technique. 400 questionnaires were distributed to the respondents to collect the data. Informed consent was obtained from respondents before collecting the data from them. In addition, respondents were informed that there are no right or wrong answers in order to reduce their tendency to provide socially desirable answers and thus reduce common method bias [69]. 38 questionnaires were incomplete, and 362 were finally used in the research, giving a valid response rate of 90.5%. A small incentive was given to each respondent for completing the survey, which took less than 7 min to complete.

Out of a total of 362 respondents, 70.2% of them are male, and 29.8% are female. The majority of the respondents have high educational qualifications. 44.2% of them completed graduation, and 18.0% completed post-graduation. Respondents aged between 18 and 35 account for 92.3% of the total respondents. 89% of respondents previously visited heritage sites, and only 11% had no heritage visit experience. More than 90% of the respondents have access to electronic payment services such as debit or credit cards or mobile financial services, implying that online payment is not a significant barrier anymore in e-commerce transactions. As most of the population does not have access to debit and credit cards, mobile financial services are popular for payment in e-commerce transactions in Bangladesh. 86.7% of respondents have access to mobile financial services (Bkash, Rocket, Nagad, etc.). 96.7% of the respondents have electronic devices that can be used to complete an e-commerce transaction. Smartphones (82.9%) dominate the device ownership category, implying that smartphone-compatible websites or applications are important requirements for promoting e-ticketing services. Table 2 presents the demographic statistics of respondents.

**Table 2:** Demographic statistics of respondents.

Variable	Category	Percentage	Variable	Category	Percentage
Gender	Male	70.2% (254)	Past visit	No visit	11.0% (40)
	Female	29.8% (108)		1–2	47.0% (170)
Education	Primary	2.2% (8)		3–4	24.6% (89)
	SSC	9.4% (34)		5–6	10.8% (39)
	HSC	26.2% (95)		Over 7	6.6% (24)
	Graduate	44.2% (160)	Access to e-payment	Credit/debit card	6.4% (23)
	Postgraduate	18.0% (65)		Mobile financial services	72.9% (264)
Age	18–25	67.4% (244)		Both of above	13.8% (50)
	26–35	24.9% (90)		None	6.9% (25)
	36–45	4.4% (16)	Access to device	Smartphone	82.9% (300)
	46–55	3.0% (11)		Tablet	1.9% (7)
	Above 55	0.3% (1)		Computer	11.9% (43)
**n = 362**				None	3.3% (12)

SmartPLS 4 software was used to analyze the data and evaluate the measurement and structural models. The PLS-based Structural Equation Modeling (SEM) technique was applied because it is less stringent on non-normal data and is recommended when the sample size is small [70]. The first run of smartPLS was activated to measure the reliability and validity of the measurement model. The reliability and validity of the measurement model were assessed by calculating Cronbach’s α, composite reliability (CR), factor loadings, and average variance extracted (AVE) [70]. Discriminant validity was also assessed. Once the evaluation criteria of the measurement model were met, the structural model was tested with a bootstrap method with 5000 resamples. Path coefficients were calculated to assess the relationship between constructs and test the hypotheses.

## 4 Results

### 4.1 Evaluation of measurement model

This study used the variance inflation factors (VIFs), outer loadings, internal reliability, CR, and discriminant validity measures to evaluate the measurement model. The first run of the smartPLS was used to check whether the VIF values of all the indicators were lower than the acceptable threshold of 5 [71]. Table 3 shows that the VIF values of all the indicators of the research model range between 1.309 and 2.760. The values are below the acceptable threshold, indicating no significant multi-collinearity issues. Moreover, a full collinearity test reveals that the VIF values of the inner model are lower than 3.3. Therefore, the model is considered free from common method bias [72]. Table 4 shows the collinearity Statistics (VIF) of the inner model used to assess common method bias. Then, the measurement model’s internal reliability and validity were tested. Cronbach’s α and CR were used to measure the internal consistency reliability of the measures of the constructs. Cronbach’s α, although traditionally used to measure internal consistency reliability, has some limitations. CR is a better internal consistency measure [70]. For our measurement model, Cronbach’s α and CR range between 0.709 and 0.881 and 0.712 and 0.881, respectively. The high values (above 0.7) of Cronbach’s α and CR for all constructs indicate good internal consistency reliability.

**Table 3:** Measures of reliability and validity of the measurement model.

Construct	Indicator	Mean	Standard Deviation	VIF	Loadings	Cronbach’s alpha	CR (rho_a)	CR (rho_c)	AVE
BI	BI1	3.814	0.885	1.325	0.781	0.746	0.745	0.855	0.663
	BI2	3.607	0.912	1.770	0.850				
	BI3	3.493	0.999	1.627	0.811				
ATT	ATT1	3.695	0.833	1.627	0.785	0.809	0.813	0.875	0.636
	ATT2	3.776	0.817	1.799	0.820				
	ATT3	3.789	0.852	1.583	0.771				
	ATT4	3.729	0.824	1.639	0.812				
EOU	EOU1	3.681	0.968	1.616	0.759	0.791	0.800	0.864	0.614
	EOU2	3.731	0.916	1.693	0.796				
	EOU3	3.543	0.938	1.624	0.817				
	EOU4	3.596	0.863	1.524	0.760				
PU	PU1	3.720	0.963	1.411	0.775	0.709	0.712	0.837	0.632
	PU2	3.895	0.809	1.489	0.825				
	PU3	3.953	0.770	1.309	0.784				
SN	SN1	3.612	0.871	1.493	0.892	0.730	0.731	0.881	0.787
	SN2	3.654	0.944	1.493	0.883				
PC	PC1	3.720	0.963	2.176	0.884	0.881	0.881	0.927	0.808
	PC2	3.895	0.809	2.760	0.911				
	PC3	3.953	0.770	2.633	0.902

**Table 4:** Collinearity statistics (VIF)-Inner model.

	ATT	BI	EOU	PC	PU	SN
**ATT**		1.993				
**BI**						
**EOU**	1.421	1.643			1.00	
**PC**	1.070	1.113				
**PU**	1.384	1.531				
**SN**	1.344	1.437				

The model’s convergence validity was tested using factor loadings and AVE. The factor loading of each indicator of the latent constructs was above the threshold of β = 0.7. The AVE of all the constructs exceeded the minimum threshold of 0.5, indicating convergence validity [70]. Table 3 shows the results of the reliability and validity of our measurement model.

The results (Table 3) demonstrate that the mean values of all the items of the constructs are greater than 3. The items or statements were measured by a 5-point Likert scale, where 3 was the neutral position. Any mean value above 3 indicates favorable opinions or agreement with the items. The standard deviation values of the items are less than 1, indicating that the opinions of the visitors are centered around mean values. Therefore, the mean values of the research suggest that most visitors have favorable attitudes and intentions toward adopting e-ticketing. Moreover, they perceive the ease of use and usefulness of e-ticketing, conform to the subjective norm, and feel increased privacy concerns about personal data.

Discriminant validity measures whether the constructs are empirically distinct from others or are uncorrelated. We tested discriminant validity to analyze the distinctiveness of the constructs of our model. Two methods widely used to evaluate discriminant validity are the Fornell-Larcker criterion and cross-loadings of the indicators [71]. In addition, Heterotrait-monotrait (HTMT) criterion has recently emerged as a popular method for discriminant validity assessment. According to Henseler et al. [73], the Fornell and Larcker criterion and the cross-loading do not reliably detect the lack of discriminant validity in common research situations. Therefore, the author proposed the HTMT criterion to assess discriminant validity sufficiently. This research has used all three approaches to evaluate the discriminant validity of the measurement model.

**First,** Fornell-Larcker criterion suggests that the square root of AVE in each construct should be larger than other correlations among the constructs. The results show that the square root of AVE in each construct is larger than other correlations among the constructs. Therefore, the Fornell-Larcker Criterion is not violated [74]. Table 5 presents the results of the Fornell-Larcker Criterion.

**Table 5:** Fornell-Larcker Criterion of the reflective constructs.

	ATT	BI	EOU	PC	PU	SN
**ATT**	0.797					
**BI**	0.679	0.814				
**EOU**	0.586	0.629	0.784			
**PC**	−0.314	−0.424	−0.223	0.899		
**PU**	0.545	0.42	0.462	−0.197	0.795	
**SN**	0.503	0.539	0.437	−0.182	0.421	0.887

**Second,** the values of cross-loadings are also used to assess discriminant validity. Discriminant validity is confirmed if an indicator’s loadings are larger than all of its cross-loadings [71]. The results show that the loadings of each indicator on its construct are higher than the loadings on the other constructs, indicating the discriminant validity of the model. Table 6 shows the cross-loadings of the reflective constructs.

**Table 6:** Cross-loadings of the reflective constructs.

	ATT	BI	EOU	PC	PU	SN
**ATT1**	**0.785**	0.483	0.469	−0.214	0.450	0.411
**ATT2**	**0.820**	0.533	0.452	−0.204	0.425	0.454
**ATT3**	**0.771**	0.493	0.458	−0.230	0.427	0.295
**ATT4**	**0.812**	0.643	0.489	−0.342	0.437	0.434
**BI1**	0.600	**0.781**	0.576	−0.242	0.407	0.450
**BI2**	0.570	**0.850**	0.477	−0.378	0.264	0.472
**BI3**	0.482	**0.811**	0.476	−0.425	0.352	0.392
**EOU1**	0.393	0.426	**0.759**	−0.195	0.323	0.268
**EOU2**	0.500	0.434	**0.796**	−0.138	0.392	0.369
**EOU3**	0.510	0.614	**0.817**	−0.183	0.388	0.387
**EOU4**	0.420	0.473	**0.760**	−0.185	0.338	0.334
**PC1**	−0.289	−0.386	−0.197	**0.884**	−0.196	−0.185
**PC2**	−0.294	−0.374	−0.216	**0.911**	−0.182	−0.150
**PC3**	−0.264	−0.385	−0.187	**0.902**	−0.151	−0.156
**PU1**	0.417	0.261	0.345	−0.057	**0.775**	0.310
**PU2**	0.438	0.369	0.373	−0.175	**0.825**	0.359
**PU3**	0.443	0.364	0.381	−0.224	**0.784**	0.332
**SN1**	0.487	0.458	0.394	−0.166	0.418	**0.892**
**SN2**	0.404	0.500	0.382	−0.158	0.327	**0.883**

**Third,** using the HTMT criterion as a measure of discriminant validity involves comparing it to a predefined threshold. Higher HTMT values than this threshold indicate a lack of discriminant validity. Some authors suggested a threshold of 0.85 [75,76], while others suggested a value of 0.90 [77,78]. The result shows that all HTMT values except one are below the threshold of 0.85, and all HTMT values are below 0.90, establishing the discriminant validity of the measurement model. Table 7 shows the HTMT ratio of reflective constructs.

**Table 7:** Heterotrait-monotrait ratio (HTMT) of the reflective constructs.

	ATT	BI	EOU	PC	PU	SN
**ATT**						
**BI**	0.864					
**EOU**	0.725	0.804				
**PC**	0.367	0.528	0.268			
**PU**	0.719	0.571	0.612	0.242		
**SN**	0.649	0.73	0.569	0.227	0.582	

### 4.2 Evaluation of structural model

**Fig. 2:**
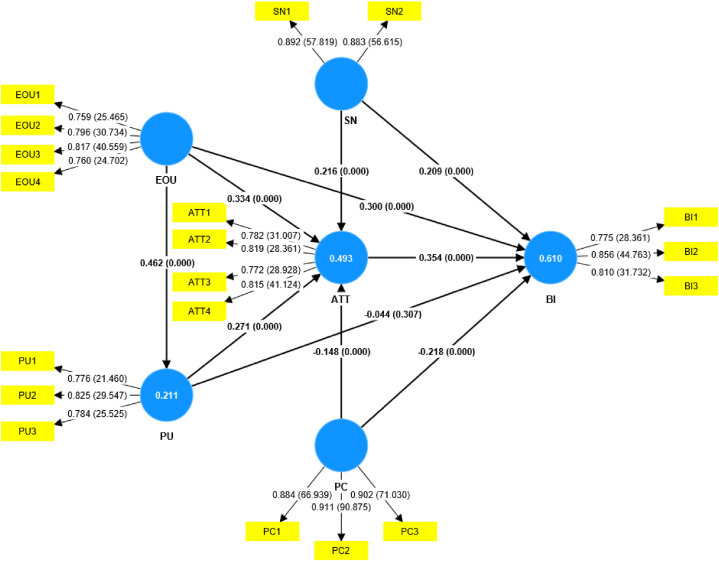
The structural model.

The quality of the structural model is assessed based on the predictive accuracy or its ability to predict endogenous constructs [70]. The adjusted R^2^ values assess the structural model’s predictive accuracy. The adjusted R^2^ values for endogenous variables, namely PU, ATT, and BI, are 0.211, 0.493, and 0.610, respectively, indicating that the model explains 21.1% of the variance in PU, 49.3% of the variance in ATT and 61% of the variance in BI. The R^2^ value of PU is considered weak, whereas the R^2^ values of ATT and BI are considered moderate [70]. Fig. 2 presents the structural relationships between the exogenous variables and endogenous variables.

Path coefficients were calculated to analyze the association between exogenous and endogenous variables and test the hypotheses. The outer model shows factor loadings and t values, whereas the inner model shows path coefficients and p values. We used the bootstrapping method with 5000 resamples to calculate path coefficients. Bootstrapping with a minimum of 5000 samples was suggested to assess the significance of path coefficients [71]. Table 8 presents the results of path coefficients and hypothesis testing.

**Table 8:** Results of path coefficients, hypothesis testing, and effect size.

Hypothesis	Latent variables	Original sample	Sample mean	Standard deviation	T statistics	P values	Decision	f-square
H1	ATT → BI	0.354	0.352	0.057	6.173	0.000	Accept	0.162
H2	EOU→ ATT	0.334	0.334	0.056	5.937	0.000	Accept	0.156
H3	EOU → BI	0.300	0.301	0.047	6.361	0.000	Accept	0.144
H4	PU → ATT	0.271	0.271	0.052	5.226	0.000	Accept	0.106
H5	PU → BI	−0.044	−0.043	0.043	1.022	0.307	Reject	0.003
H6	EOU→PU	0.462	0.464	0.054	8.569	0.000	Accept	0.271
H7	SN → ATT	0.216	0.217	0.049	4.428	0.000	Accept	0.069
H8	SN → BI	0.209	0.210	0.047	4.493	0.000	Accept	0.079
H9	PC → ATT	−0.148	−0.148	0.041	3.653	0.000	Accept	0.041
H10	PC → BI	−0.218	−0.217	0.035	6.138	0.000	Accept	0.110

*Notes*: f-square ≥ 0.02 small effect; f-square ≥ 0.15 medium effect; f-square ≥ 0.35 large effect.

Results show that ATT is positively associated with BI. The relationship is significant with a medium effect. EOU has a significant positive association with ATT, BI, and PU with medium effects. PU has a significant positive association with ATT with a small effect. However, the association between PU and BI is not significant. SN is positively associated with ATT and BI. The associations are significant with small effects. On the other hand, PC has a negative association with ATT and BI. The associations are significant with small effects. The relationships between exogenous and endogenous variables are significant at a 0.01 level of significance.

In sum, all the exogenous variables except PU are significantly associated with ATT and BI. PU is significantly associated with ATT but not with BI. Only PC is negatively associated with ATT and BI. All other relationships between variables that are significant at a 0.01 level of significance are positive.

### 4.3 Mediation analysis

This research satisfies the following requirements of mediation analysis to analyze the mediating role of ATT in the structural model [79]:

a) The mediator (ATT) is significantly related to the dependent variable (BI).
b) Independent variables (EOU, SN, PC) are significantly related to the mediator (ATT).
c) Independent variables (EOU, SN, PC) are significantly related to the dependent variable (BI).

Therefore, mediation analysis was performed to assess the mediating role of ATT in the relationship between EOU and BI, PC and BI, and SN and BI, and test 3 additional hypotheses:

H11 Attitude mediates the relationship between ease of use and e-ticketing intention.

H12 Attitude mediates the relationship between subjective norm and e-ticketing intention.

H13 Attitude mediates the relationship between privacy concerns and e-ticketing intention.

Table 9 presents the results of the mediation analysis. The results show that the total effects of EOU, SN, and PC on BI are significant. When the ATT is included as a mediator, significant indirect effects of EOU, SN, and PC on BI are found. In addition, the direct effects of EOU, SN, and PC on BI still remain significant, as shown in Table 8. Therefore, it is demonstrated that ATT partially mediates the relationship between EOU and BI, SN and BI, and PC and BI [80].

**Table 9:** Results of mediation analysis.

Total effects						
Latent variables	Original sample	Sample mean	Standard deviation	T statistics	P values	Decision
ATT→BI	0.354	0.352	0.057	6.173	0.000	Significant
EOU→ATT	0.459	0.460	0.049	9.443	0.000	Significant
EOU→BI	0.442	0.443	0.041	10.741	0.000	Significant
PU→ATT	0.271	0.271	0.052	5.226	0.000	Significant
PU→BI	0.052	0.052	0.048	1.080	0.280	Not significant
EOU→PU	0.462	0.464	0.054	8.569	0.000	Significant
SN→ATT	0.216	0.217	0.049	4.428	0.000	Significant
SN→BI	0.286	0.287	0.043	6.578	0.000	Significant
PC→ATT	−0.148	−0.148	0.041	3.653	0.000	Significant
PC→BI	−0.270	−0.269	0.036	7.427	0.000	Significant
**Specific indirect effects**						
**Indirect path**	**Original sample**	**Sample mean**	**Standard deviation**	**T statistics**	**P values**	**Decision**
H11: EOU→ATT→BI	0.118	0.117	0.027	4.366	0.000	Partial mediation
H12: SN→ATT→BI	0.076	0.077	0.022	3.449	0.001	Partial mediation
H13: PC→ATT→BI	−0.052	−0.052	0.017	3.145	0.002	Partial mediation

## 5 Discussion

This research has investigated the extent to which ATT, EOU, PU, SN, and PC constructs are directly and indirectly associated with heritage visitors' intention to use the e-ticketing system. EOU, PU, ATT, and BI were taken from TAM [15,39,40]. SN and ATT were taken from TPB [25,26]. PC was incorporated as an additional exogenous construct in the proposed model.

The results of our research show that ATT is significantly and positively associated with BI. Therefore, H1 is accepted. The results imply that a positive attitude towards the e-ticketing system is essential for adopting e-ticketing by heritage visitors. This result is supported by findings of previous studies [22,81]. This research has also identified factors contributing to a positive attitude toward e-ticketing.

EOU is significantly and positively associated with ATT and BI. Therefore, H2 and H3 are accepted. Moreover, ATT partially mediates the relationship between EOU and BI. Therefore, H11 is also accepted. If visitors perceive the e-ticketing system as easy to operate, they form a positive attitude toward it and intend to use it. The positive association between EOU and ATT is supported by previous studies [81,82]. The positive association between EOU and BI is also supported by Bhatiasevi and Yoopetch [83] and Ahn et al. [84] but contradicted by Marquez et al. [35].

PU also has a significant positive association with ATT. Therefore, H4 is accepted. This result is supported by the findings of previous studies by Chen et al. [81] and Hossain et al. [82]. However, the association between PU and BI is not significant. Therefore, H5 is not accepted. The results indicate that visitors who perceive the usefulness of e-ticketing form a positive attitude toward e-ticketing. But, the visitors' perceived usefulness does not directly cause the visitors to use the e-ticketing system but through forming a positive attitude toward e-ticketing. The result that no significant association was found between PU and BI contradicts Marquez et al. [35] and Ahn et al. [84]. One possible explanation for this result is that visitors are resistant to changing their habits of getting entry tickets from the ticket counter.

There is a significant positive association between EOU and PU. Therefore, H6 is accepted. This result indicates that visitors' perception of the e-ticketing technology as easy to use or operate causes them to perceive e-ticketing as useful. The direct and indirect associations between SN and ATT and between SN and BI are significant. Therefore, H7, H8, and H12 are accepted. The significant positive direct association indicates that when visitors perceive that their close associates will encourage their use of the e-ticketing system, they form a positive attitude toward e-ticketing and intend to use it, respectively. SN is also indirectly related to BI; ATT mediates the relationship between SN and BI. Previous studies support the association between SN and BI [1,22,81].

The direct and indirect relationships between PC and BI are also significant. Therefore, H9, H10, and H13 are accepted. The negative direct associations between PC and ATT and between PC and BI indicate that visitors with high privacy concerns have negative attitudes toward e-ticketing and are less likely to use the e-ticketing system when there are alternatives. The significant negative indirect association between PC and BI indicates that privacy concerns reduce the intention to use the system by developing a negative attitude toward the e-ticketing system. Previous research by Liang and Shiau [1] supports the negative relationship between PC and BI.

## 6 Theoretical and practical implications and conclusion

The findings of the research help academics and practitioners alike understand the needs and concerns of heritage visitors about the use of the e-ticketing system from the perspective of a developing country. This research contributes to literature relating to TAM by integrating subjective norms and privacy concerns into the original model to explain visitors' e-ticketing adoption behavior in the heritage tourism context. Privacy concerns of visitors are often neglected in developing countries; however, this research provides evidence that visitors' privacy concerns are associated with attitudes towards e-ticketing technology and behavioral intentions to adopt e-ticketing technology. Therefore, heritage visitors' privacy concerns should be considered an important predictor of e-ticketing and other self-service technology adoption in the heritage tourism sector. This research demonstrates that subjective norm influences heritage visitors' attitudes and intentions to use the e-ticketing system. The findings imply that visitors' concern for privacy and other people’s opinions, in addition to ease of use and perceived usefulness of technology, are associated with visitors' cognitive processes-attitudes and decision-making of adopting the e-ticketing system.

This research has extended the application of the technology adoption model to the heritage tourism sector in a developing country and minimized our knowledge gaps of heritage visitors' technology acceptance behavior. The research has practical implications for destination managers and other tourism practitioners. The findings suggest that the authorities of heritage sites take the initiative to develop a positive attitude of their existing and potential visitors towards the e-ticketing system to increase the behavioral intention to adopt the e-ticketing system. Developing a user-friendly e-ticketing system and communicating its benefits can help improve the visitors' intention to use the e-ticketing system. Perceived ease of use can be enhanced by training through short video materials or written guidelines on how to use the system.

Privacy concerns of heritage visitors should not be ignored. Mitigating the privacy concerns of visitors can help develop a positive attitude toward the e-ticketing system and increase its adoption. The adoption of strict policies on consumer data privacy may help enhance consumer confidence and mitigate consumers' perceived risk of data leakage. Moreover, sharing with the visitors the statistics on the worldwide acceptance and popularity of e-ticketing systems can enhance visitors' perceived social acceptance and confidence that they belong to a larger community that uses and supports the use of e-ticketing systems worldwide.

This research acknowledges a few limitations. The majority (over 90%) of respondents in this research are aged between 18 and 35. The low participation of respondents of other age groups (over 35 years old) happened because of their low visitation rate and unwillingness to participate in the study. A few probable reasons for the low visitation rate of the older population are lack of facilities, poor accessibility, overcrowding, and lack of promotional activities targeted toward them. Therefore, caution should be taken to generalize the findings of the research. Future research on samples representing all age groups is recommended to increase the generalizability of the results. Moreover, a longitudinal study is needed to investigate the relationship between behavioral intention to use the e-ticketing system and actual behavior.

## Author contribution statement

Md Islam: Conceived and designed the experiments; Performed the experiments; Analyzed and interpreted the data; Contributed reagents, materials, analysis tools or data; Wrote the paper.

## Funding statement

This research did not receive any specific grant from funding agencies in the public, commercial, or not-for-profit sectors.

## Data availability statement

Data will be made available on request.

## Declaration of interest’s statement

The authors declare no competing interests.
