# Supplementary material for: Factors affecting adoption of self-service E-ticketing technology: A study on heritage sites in Bangladesh
Source: Heliyon. 2023 Mar 20;9(3):e14691. doi: 10.1016/j.heliyon.2023.e14691 (PMC10033747; doi:10.1016/j.heliyon.2023.e14691)
Supplement: Multimedia component 1 [file mmc1.docx]

**E-ticketing at heritage sites in Bangladesh**

**Consent form**

This research titled Visitors’ intention to adopt self-service E-ticketing system for heritage sites’ visit in Bangladesh is being conducted by a faculty member at Begum Rokeya University. We will ask you some questions about your intention to use the e-ticketing system for heritage tours. This questionnaire will take approximately 5-10 minutes to complete. You can stop answering anytime for any reason. The data collected from you will be confidential and will not be used for any other purpose than this research. Please provide your consent below if you are willing to participate in this survey.

**Do you want to participate in the survey?**

1) Yes

2) No

**Demographic profile**

I. Gender

1) Male

2) Female

II. Education

1) Primary

2) SSC

3) HSC

4) Graduate

5) Postgraduate

III. Age

1) 18-25

2) 26-35

3) 36-45

4) 46-55

5) >55

IV. Number of past visits to heritage sites

1) No visit

2) 1-2

3) 3-4

4) 5-6

5) Over 7

V. Which digital payment services do you have access to?

1) Credit card/debit card

2) Mobile financial services (Bkash, Rocket, Nagad, etc.)

3) Both credit/debit card and mobile financial service

4) None

VI. Which device do you use?

1) Smartphone

2) Tablet

3) Computer (laptop, desktop)

4) No

**Research questions**

**Intention**

A) I intend to use the E-ticketing system of heritage sites to buy entry tickets in the future

1. Strongly disagree
2. Disagree
3. Neutral
4. Agree
5. Strongly agree

B) I plan to purchase an online ticket for my heritage site visit in the next 6 months

1. Strongly disagree
2. Disagree
3. Neutral
4. Agree
5. Strongly agree

C) I will use E-ticketing for my heritage site visit in the next 6 month

1. Strongly disagree
2. Disagree
3. Neutral
4. Agree
5. Strongly agree

**Attitude**

A) E-ticketing system is “enjoyable”

1. Strongly disagree
2. Disagree
3. Neutral
4. Agree
5. Strongly agree

B) E-ticketing system is “Pleasant”

1. Strongly disagree
2. Disagree
3. Neutral
4. Agree
5. Strongly agree

C) E-ticketing system is “Good”

1. Strongly disagree
2. Disagree
3. Neutral
4. Agree
5. Strongly agree

D) E-ticketing system is “Useful”

1. Strongly disagree
2. Disagree
3. Neutral
4. Agree
5. Strongly agree

**Ease of Use**

A) It would be easy to learn the E-ticket buying process

1. Strongly disagree
2. Disagree
3. Neutral
4. Agree
5. Strongly agree

B) It would be easy to buy an E-ticket for heritage site visits

1. Strongly disagree
2. Disagree
3. Neutral
4. Agree
5. Strongly agree

C) An E-ticket system would be understandable

1. Strongly disagree
2. Disagree
3. Neutral
4. Agree
5. Strongly agree

D) An E-ticketing system would be flexible for buying entry tickets for heritage sites

1. Strongly disagree
2. Disagree
3. Neutral
4. Agree
5. Strongly agree

**Perceived usefulness**

A) An E-ticketing system in heritage sites would help buy tickets more quickly

1. Strongly disagree
2. Disagree
3. Neutral
4. Agree
5. Strongly agree

B) E-ticketing in heritage sites would make it easier to buy the ticket

1. Strongly disagree
2. Disagree
3. Neutral
4. Agree
5. Strongly agree

C) E-ticketing system in heritage site would be useful for me in my future visit (tour)

1. Strongly disagree
2. Disagree
3. Neutral
4. Agree
5. Strongly agree

**Subjective norms**

A) Most people (who are important to me) will approve (support/ encourage) of my using an

Electronic-ticketing system

1. Strongly disagree
2. Disagree
3. Neutral
4. Agree
5. Strongly agree

B) Most people (who are important to me) would not complain if I buy E-ticket of heritage sites

1. Strongly disagree
2. Disagree
3. Neutral
4. Agree
5. Strongly agree

**Privacy concerns**

A) I am anxious about the consequences of sharing my information with an E-ticketing system on a

heritage site

1. Strongly disagree

2. Disagree

3. Neutral

4. Agree

5. Strongly agree

B) It is important to me that the heritage site protects my identity in the E-ticketing system

1. Strongly disagree

2. Disagree

3. Neutral

4. Agree

5. Strongly agree

C) I believe my personal information would be well-protected in the E-ticketing system on the

heritage site

1. Strongly disagree

2. Disagree

3. Neutral

4. Agree

5. Strongly agree

Thanks
